# The Impact of Social and Cultural Engagement and Dieting on Well-Being and Resilience in a Group of Residents in the Metropolitan Area of Naples

**DOI:** 10.1155/2016/4768420

**Published:** 2016-05-19

**Authors:** Antonio Rapacciuolo, Pasquale Perrone Filardi, Rosario Cuomo, Vincenzo Mauriello, Maria Quarto, Annamaria Kisslinger, Gianluigi Savarese, Maddalena Illario, Donatella Tramontano

**Affiliations:** ^1^Department of Advanced Biomedical Science, University of Naples Federico II, 80131 Naples, Italy; ^2^Department of Clinical Medicine and Surgery, University of Naples Federico II, 80131 Naples, Italy; ^3^Arci Movie, 80147 Naples, Italy; ^4^Department of Physics, University of Naples Federico II, 80131 Naples, Italy; ^5^IEOS-CNR, Naples, Italy; ^6^Fondazione GENS Onlus, 80121 Naples, Italy; ^7^Department of Translational Medical Sciences, University of Naples Federico II, 80131 Naples, Italy; ^8^R&D Unit, Federico II University Hospital, 80131 Naples, Italy; ^9^Department of Molecular Medicine and Medical Biotechnology, University of Naples Federico II, 80131 Naples, Italy

## Abstract

Social isolation and exclusion are associated with poor health status and premature death. A number of related isolation factors, inadequate transportation system and restrictions in individuals' life space, have been associated with malnutrition in older adults. Since eating is a social event, isolation can have a negative effect on nutrition. Cultural involvement and participation in interactive activities are essential tools to fight social isolation, and they can counteract the detrimental effects of social isolation on health. To provide data supporting the hypothesis that encouraging participation might represent an innovative preventive and health promoting strategy for healthy living and aging, we developed an* ad hoc* questionnaire to investigate the relationship between cultural participation, well-being, and resilience in a sample of residents in the metropolitan area of Naples. The questionnaire includes a question on adherence to diet or to a special nutritional regimen; in addition, the participants are asked to mention their height and weight. We investigated the relationship between BMI, adherence to diet, and perceived well-being (PWB) and resilience in a sample of 571 subjects over 60 years of age. Here, we present evidence that engagement into social and cultural activities is associated with higher well-being and resilience, in particular in females over 60 years of age.

## 1. Introduction

Social isolation and exclusion are associated with poor health status and premature death, while social cohesion, the quality of social relationships and the existence of trust, mutual obligations, and respect in communities, helps to protect people and their health. Since social and family relationships are embedded within the definition of a “good quality of life” for all age groups, and particularly for older adults, it is social isolation inversely correlates with well-being [1–5]. A number of social isolation factors, inadequate transportation system and restrictions in individuals' life–space, have been associated with poor nutrition in older adults. Good nutrition is important for health and well-being at all stages of the life course; however, its determinants change with age. Older adults are particularly prone to slipping into a pattern of an inadequate diet because of decreased mobility associated with physical disabilities and/or fewer financial resources to spend on food [6, 7]. Moreover, past evidence support that socially isolated older adults are at a greater risk of dietary inadequacy because they lack social support, which promotes good diet. In recent years, several studies focused on the relationship between cultural access and physical and psychological health. Their results suggest that participation in social and cultural activities is beneficial for health, since it helps people to remain active and socially connected, avoiding social isolation and loneliness [8, 9]. In elderly people, participation in social and cultural activities correlates with decreased medication consumption and hospitalization. The association between cultural activities and health outcomes has been analyzed in the medical field, in the context of mental health, cognitive decline, onset of dementia, and related disorders [10–16].

Moreover, data are growing in support of the relationship between cultural and social engagement and well-being [17–23]. Well-being is shaped by not only the absence of disease and reduced physical functioning, but also by the presence of positive physical, mental, and psychosocial state. In this view, well-being is crucial to many aspects of our daily lives, since it includes global judgments such as emotions and resilience, quality of relationships, and overall life satisfaction [24–27]. In particular, cultural participation is the second predictor of psychological well-being after (presence/absence of) major diseases, and in this respect, it has a significantly stronger impact than variables such as income, place of residence, age, gender, or occupation. Finally, links have been documented between well-being and multiple aspects of physical health and mortality, cardiovascular disease, biological risk factors for infectious diseases, dementia, and disability in later life [28–31]. Considering the close relationship of high well-being with key health outcomes, tracking and improving well-being is becoming increasingly important for global organizations, governments, companies, and communities worldwide [31, 32].

Therefore, recently a number of studies explored losses in well-being caused by 2008 economic crisis. The findings reveal the negative impact of GDP fall, unemployment rising, and banking crashes on subjective and psychological well-being [33–38]. In addition, several reports provide evidence of an increased prevalence of suicides because of the recent great recession [39–41].

Finally, according to the United Nations Interregional Crime and Justice Research Institute, the global economic crisis has disproportionate effects on women [42].

The average Italian household has been severely affected by the crisis, with impacts that are particularly visible when looking at household income, jobs, life satisfaction, and civic engagement. From 2007 to 2011, Italy recorded a cumulative decline in real household disposable income of around 7%, one of the largest declines among the OECD countries. Market income inequality (before taxes and transfers) increased by 2% between 2007 and 2010, well above the OECD average of 1.2%. The largest impact of the crisis on people's well-being has come through lower employment and deteriorating labour market conditions. Between 2007 and 2012, the employment rate decreased by more than 1 percentage point in Italy, while the long-term unemployment rate increased by almost 3 percentage points. The poor employment situation had a major impact on life satisfaction. From 2007 to 2013, the percentage of Italian people declaring being very satisfied with their lives fell from 58% to 40% [42]. Moreover, according to data from the Italian Institute of Statistics (ISTAT), the crisis have worsened both the north–south and the gender gap in in terms of life satisfaction. The data indicate that in Italy the males are on average more satisfied of their life than females (M 36% > F 34%) and in addition that both males and females living in the north, that is, Lombardy region, are more satisfied of their life than those living in the south, that is, Campania region, (Lombardy: males 42%, females 41%; Campania: males 21,9%, females 19,4%). The 2008 crisis has deeply affected the city of Naples, the capital of the Campania region, and its metropolitan area worsening both the north > south gap and the chronic structural local problems. In that, the report of the Italian Institute of Statistics (ISTAT) shows that in 2014 Naples ranks 101 over the 107 Italian province in terms of quality of life. In addition, ISTAT reports that in 2014 the unemployment rate in Italy was 12,4% and in Naples 24,26% and that in the same year employment rate was slowly growing in Northern (+0,4%) and Central (+1,8%) Italy, while further declining in the South (−0,8%, −45.000 units) [43].

Long-lasting progressive and strong deindustrialization, high level of unemployment, and a large influx of illegal immigrants had explosive consequences on the breakdown of the social fabric that from the specific suburbs spreads like wildfire to the entire city of Naples. At present, local degradation and impoverishment, overlapping with welfare cut, consequent to nation-wide crisis, make day-by-day life difficult, in particular for the more fragile part of the population such as the elderly people living in the metropolitan area of Naples. On the other hand, Naples and its surrounding areas display an extraordinary richness of both tangible and intangible cultural heritage. The value of Naples monuments building, ancient ruins together with its location on the Mediterranean sea, gained the city to be listed by Unesco as a World Heritage Site in 1995 (http://whc.unesco.org/en/list/726/). Moreover, a number of artists, actors, directors, writers, and gallerists, some of them well-recognized world-wide, struggle every day to keep the city long history of creativity alive that represents the worldwide recognized Naples intangible heritage.

In this scenario, we considered the investigation of how citizens of the Metropolitan area of Naples react to adversities and how and if cultural tangible and intangible heritage would influence their subjective well-being valuable. The oldest-old are the fastest-growing sector in society, due to life expectancy increases and improved treatments for life-threatening diseases. Understanding the determinants of psychological well-being and their relationship with health outcomes at older ages is particularly important, since a high proportion of the budget for health and social care is devoted to the care of older people.

Due to the particularly high ageing index (A.I. = 120,3 in 2014) [43] and to the economic difficulties of the Metropolitan area of Naples, it is important to consider new affordable tools and strategies to promote a healthy ageing and to face the “burden” of this demographic change.

On these bases, we decided to take a snapshot of the metropolitan area of the city of Naples investigating the relationship between adherence to diet or nutritional regimen, BMI, and subjective well-being and the impact of social and cultural participation. In particular, we focused on the population over 60 years of age and on gender difference. To our knowledge, this is the first survey investigating subjective well-being in the metropolitan area of the city of Naples.

## 2. Methods

Within the framework of the A3 Action Group of the European Innovation Partnership on Active and Healthy Ageing and of the “Getting Optimize Aging Life Quality” (GOAL) project, Fondazione GENS Onlus developed an* ad hoc* anonymous questionnaire to assess perceived well-being, resilience, and perceived health and their relation with engagement into social and cultural experiences. The questionnaire comprises the following sections:

- Sociodemographic information, age, sex, place of birth, education, employment, and marital status.
- Psychological well-being: investigated by means of Psychological General Well-Being-Short (PGWB-S) questionnaire developed and validated in the Italian version by Grossi and coworkers in 2006 [44]. Grossi and coworkers reduced the number of items from the original 22-item PGWBI to 6 items to achieve a higher acceptability of the questionnaire in the population, to shorter time of administration and to obtain a better response rate together with lower rate of missing data. The authors reported that PGWB-S 6 showed that the PGWB-S maintained validity, reliability, and good acceptability for the use in various settings in Italy [44]. PGWB-S 6-item questionnaire analyzes the following domains: Anxiety, Vitality (positive), Depressed Mood, Self-Control, Positive Well-Being, and Vitality (negative) on a 0 to 5 scale referring to the four weeks before the date of the survey [44].
- Resilience according to Connor-Davidson resilience scale CD-RISC2 2 items: item 1 (“able to adapt to change”) and item 8 (“tend to bounce back after illness or hardship”) on a scale from 0 to 4 [45].
- Extent of social network.
- Participation in cultural and social activities.
- Life-style habits, PC use, smoke, diet, physical activity, transportation, number and type of diagnosed diseases, and self-reported perceived health status.

The anonymous questionnaire was submitted to volunteer participants covering wealthy, middle class, and poor neighborhoods of the metropolitan area of Naples. Trained GENS personnel explained the questionnaire and assisted volunteer participants while filling the questionnaire.

### 2.1. Statistical Analysis

Descriptive statistics were computed for all the indicators analyzed. Student's *t*-test and ANOVA with Bonferroni correction were performed to test continuous variables. Chi-square was used to test categorical variables. Linear regression analysis was performed to test the relationship between two variables. All statistical analysis were performed using Stata software (Stata Corp., College Station, TX, USA).

## 3. Results

### 3.1. Analysis of the Sample Population

Within 2014, we have collected 571 questionnaires of subjects over 60 years of age and this sample population is the object of the present work. Mean age of the 571 subjects over 60 years of age (from now on >60) is 70,05 ± 6,94 years. The >60 sample consists of 285 males and 286 females with a mean age of 70,35 ± 6,923 and 69,78 ± 6,980 years, respectively. The Subjective Well-Being (SWB) was assessed by measuring both psychological well-being and resilience. Self-reported psychological well-being referred to the past 4 weeks according to 6-item PGWB-S analyzing the following domains: Anxiety, Vitality (positive), Depressed Mood, Self-Control, Positivity, and Vitality (negative) on a 0 to 5 scale [45]. In physics, the term “resilience” indicates the power or the ability of a material to return to the original form, position, and so forth, after being bent, compressed, or stretched, and also elasticity. In the health field, resilience applies to the ability to adapt to changes and to readily recover from stressful situation like illness, depression, adversity, or the like. Thus, resilience is a key part of SWB. Resilience was assessed according to Connor-Davidson resilience scale, 2-item CD-RISC2, on a scale of 0 to 4 [45]. According to Chassany et al. and Grossi et al., PGWB scores have been grouped into the following divided categories: 0–60 Severe Distress, 61–71.0 Moderate Distress, 72–92 No Distress, and 93–110 Positive Well-Being [46, 47]. PWB score for all 571 >60 subjects was 68,22 ± 19,71, falling in the area of Moderate Distress. Males and females differently contribute to the PWB score of the whole >60 group, where the PWB score for >60 males is 71,61 ± 18,83 while that for >60 females is 64,92 ± 20,11 (*P* < 0,0001). Our results indicate that the PWB score of >60 males falls borderline between the area of No Distress and Moderate Distress, while that of the >60 female population falls within the area of Moderate Distress. It is interesting to note that PWB score of both >60 males and females, living in metropolitan area of the city of Naples, is largely below that reported for males and females living in Northern and Central Italy and in particularly in the city of Milan [47, 48]. To get a better insight in the PWB score gender difference, we analyzed the six PWB dimensions separately in males and females. The results reported in Table 1 show that females score is lower than that of males in all dimensions but Vitality (positive). On the other hand, resilience score of the whole >60 group was 5,867 ± 1,687, which resulted to be similar in >60 males and females, 5,91 ± 1,57 and 5,84 ± 1,8 respectively. However, when we analyzed resilience item 1 and item 2 separately, it came out that item (1) “able to adapt to change” and item (2) “tend to bounce back after illness or hardship” differently contribute to the cumulative resilience score. In particular, both males and females >60 are less “able to adapt to change” (resilience item 1 score, females 2,71 ± 1,08 and males 2,728 ± 1,03) but “tend to bounce back after illness or hardship” more easily (resilience item 2 score, females 3,15 ± 0,9710 and males 3,20 ± 0,87). The difference between item 1 and item 2 score resulted to be statistically significant (*P* < 0,0001) both in >60 males and females. Finally, our data indicate a correlation between resilience and PWB (*R* 0,4708, *R* square 0,2217, *P* value < 0,0001). Same correlation was observed when >60 females and males were analyzed separately (F = *R* 0,418, *R* square 0,1810, *P* value < 0,0001; M = *R* 0,545, *R* square 0,2891, *P* value (two-tailed) < 0,0001).

### 3.2. Body Mass Index

**Figure 1:**
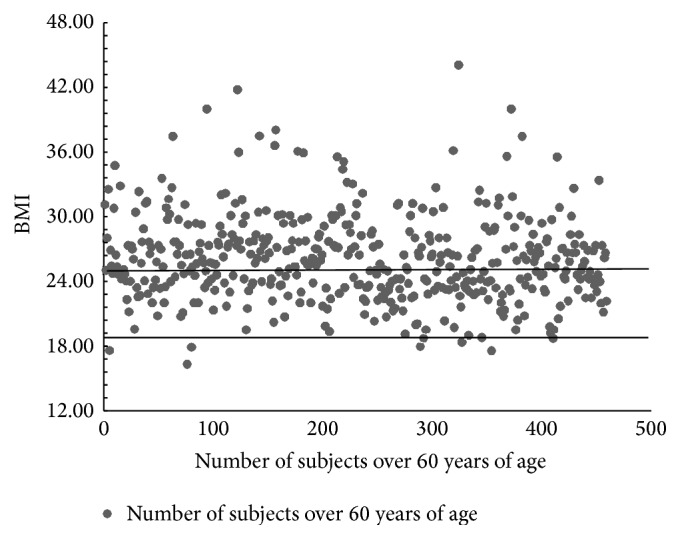
BMI values of all subjects over 60 years of age examined in the study.

Within the section related to perceived health status, participants indicated their weight and height. Body mass index (BMI), computed by dividing weight in kilograms by height in meters squared, was categorized according to WHO guidelines, underweight: BMI less than 18.5 kg/m^2^; normal weight: BMI 18.5–24.9 kg/m^2^ (reference category); overweight: BMI 25–29.9 kg/m^2^; obesity: BMI 30–40+ kg/m^2^ [49]. All subjects were requested to indicate weight and height. Mean BMI for all subjects resulted to be 25.58 ± 4.20 which falls in the range of overweight, according to the NIH indication [49]. BMI distribution of the all >60 subjects is depicted in Figure 1, and it indicates that 60% of the >60 subjects fall within the overweight and obesity category, 40% are in the range of normal weight, and only 1% are underweight.

**Figure 2:**
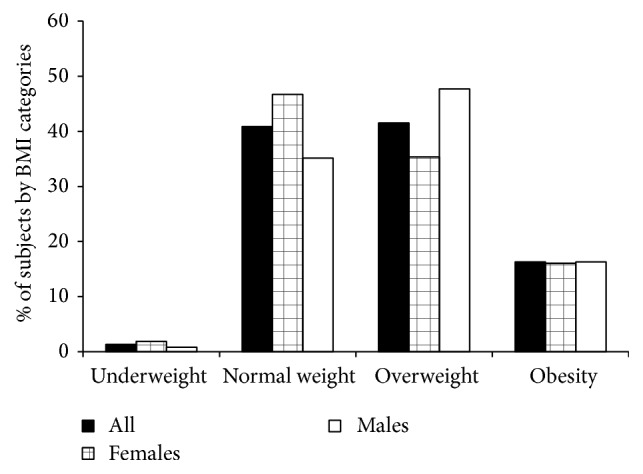
Distribution of >60 all, females, and males by BMI categories. Percentage per each group has been calculated over the total number of >60 years of age in all, females, and males groups separately.

Then we examined all females and males distribution BMI classes and the results indicated that 51% of the >60 female subjects and 64% of the >60 male subjects fall in the range of overweight and obesity (Figure 2) while 47% of female and 35% of male subjects were in the range of normal weight. Since the relation between obesity and psychological and subjective well-being is becoming a hot issue, in both the health and the economic field, we compared the BMI, well-being, and resilience in the obese group versus the normal weight one. As it is shown in Table 2(a), in all the >60 subjects, there was no significant difference in PWB and resilience score according to BMI categories. On the other hand, when we analyzed women and men separately, we found that both PWB and resilience decrease in >60 obese females with respect to normal weight group, while BMI increases (Table 2(b)). Also, in >60 males, PWB and resilience score are almost superimposable in both the normal weight and the obesity group (Table 2(c)), while BMI increases. In addition, the opposite trend of PWB and resilience score between obese males and females amplifies the gender difference that remained significant (*P* < 0,05).

Moreover, correlation analysis between BMI and PWB and resilience indicates no correlation in all the population (BMI > PWB = *R* 0,049, *R* square 0,002, *P* value = 0,299; BMI > resilience = *R* 0,056, *R* square 0,003, *P* value 0,231) and in the male population (BMI > PWB *R* 0,085, *R* square 0,007, *P* value = 0,191; BMI > resilience = *R* 0,114, *R* square, 0,013, *P* value 0,081). On the contrary, in the female population a significant correlation was found both between BMI and PWB and between BMI and resilience (BMI > PWB = *R* 0,171, *R* square 0,029, *P* value 0,012; BMI > resilience = *R* 0,173, *R* square 0,029, *P* value 0,011).

Subjects indicate eventual diagnosed disease/s within the following list of diseases: diabetes, respiratory diseases, skin diseases, gastritis, anemia, depression, osteoporosis, kidney diseases, migraine, anxiety, heart failure, arrhythmias, ischemic heart diseases, cancer, allergy, arthrosis, myocardial infarction, hypertension, obesity, liver disease, back pain, and colitis. The frequency of diabetes, hypertension, obesity, cardiovascular diseases (CVD), comprising heart failure, arrhythmias, ischemic heart disease, and myocardial infarction and depression, categorized according to BMI classes, is shown in Table 3. Hypertension resulted to be the most reported diagnosed disease within normal weight, overweight, and obesity classes in >60 females, while CVD was the most frequently reported by the >60 male group.

### 3.3. BMI, Resilience, and PWB in >60 Females and Males Participating and Nonparticipating in Cultural and Social Activities

Among the 571 >60 subjects, 78,45% are engaged into cultural and social activities, while 21,54% are not. Within the P population 52% are women and 48% are men, while in the NP population 57% are women and 43% are men. Interestingly, when we compared BMI of subjects participating (P) and nonparticipating (NP) to cultural and social activities, we observed that BMI was higher in females NP versus females P (<0,05) (Table 4(b)). On the other hand, no difference in BMI is observed between >60 males P and NP. More importantly, the >60 NP population displays PWB and resilience score significantly lower the >60 P. In particular, the >60 P male population frankly falls into the area of positive well-being, while that of NP goes in the area of moderate distress (Table 4). As for women, the PWB of the NP population dramatically crashes in the area of severe distress. These observations show an association between participation in cultural and social activities and subjective well-being, by means of PWB and resilience score. In addition, in the case of the female group, and in particular the NP females, we observed an inverse relation between BMI and PWB and resilience, since BMI increases while PWB and resilience decrease. The last observation suggests an intriguing and apparently new association between BMI, subjective well-being indicators, and participation in cultural and social activities.

### 3.4. Adherence to a Diet or a Nutritional Regimen

We then analyzed answers of >60 subjects to the question: Do you follow a diet or a nutritional regimen? The results indicate that 35% of males > 60 and 43% of females > 60 follow a diet or a nutritional regimen. Thereafter, we investigated the relation between perceived well-being and resilience and adherence to diet or nutritional regimen. Interestingly, women P adhering to diet display significantly higher PWB (*P* = 0,013) and resilience (*P* = 0,043) than the NP following diet (Table 5). In addition, a significant difference was observed in PWB and resilience between >60 female P and NP (*P* < 0,0001) nondieting. On the other hand, while PWB was higher in the >60 males of the P group with respect to the NP following a diet, resilience was similar in the two groups (Table 5). In addition, differently from females, NP males who do not follow a diet or nutritional regimen apparently are overall “happier” then NP following a diet. We then analyzed obese females and males dieting and nondieting. The results show that obese females dieting present both PWB and resilience scores higher than the nondieting obese females (PWB 61,69 ± 17,26 > 54,72 ± 16,69, *P* < 0,009; resilience 6 ± 1,57 > 5,429 ± 1,4, *P* < 0,003). Conversely, obese males dieting show both PWB and resilience scores lower than the nondieting obese males (PWB 67,35 ± 16,43 > 81,53 ± 17,18, *P* < 0,05; resilience 5,842 ± 1,46 > 6,706 ± 1,44, *P* < 0,05).

## 4. Discussion

To our knowledge, this is the first assessment of PWB and resilience conducted in the Metropolitan area of Naples. Our data show that a sample of 571 subjects over 60 years of age resident in the Metropolitan area of Naples display a PWB score of 68,22 ± 19,71 on a scale of 0 > 110, largely below PWB scores previously reported for the Italian population. In particular, Grossi et al. [47–50] reported a PWB score of 77.76 (17.73 SD) for the Italian population (1500 subjects) in 2011. The PWB score was geographically distributed as follows: north (696 subjects) 79.34 (17.71 SD), centre (293 subjects) 78.04 (17.12 SD), south (511 subjects) 75.47 (17.91 SD). Moreover, in 2013 the same authors reported that a sample of 1000 citizens of Milan displayed a PWB score of 82, 14 (15.63 SD) while that of the population over 60 years was 80,39.

The questionnaires analyzed here have been collected within 2014 when, as reported above [43], the Metropolitan area of Naples was still suffering for the economic crisis. Our results are in agreement with those reporting a relationship between unemployment and low level of PWB. Thus, we cannot exclude that the low PGWB score of this sample population of residents in the Metropolitan area of Naples reflects the detrimental effects of the economic and social crisis at local level. In particular, of note 50% of the 571 subjects over 60 years of age are retired, and retirement benefits represent for most families in the area, in a time of high unemployment, the only income to count on.

When PWB score was measured in >60 males and females separately, a gender difference was observed. It is generally reported that women have a score higher than men do in happiness, when happiness is measured as life satisfaction. It is also reported that the advantage of women in terms of happiness and life satisfaction is not uniform along the life cycle: women are less happy than men before the age of 18, happier than men until their fifties, and less happy again thereafter [51]. Moreover, the “paradox of declining female happiness” seems to indicate that the traditional gender gap in happiness (in favour of women) is progressively shrinking since the 1970s in spite of the type of technological progress, civil liberties, and gender-conscious policies that characterize modern Western societies [52].

On the other hand, it is well recognized that women score lower than men do, on measures that capture short-term positive and negative emotions and are more subject to depression symptoms [51].

The gender difference observed in PWB score in the sample of women >60 analyzed here is in line with results already reported by Grossi et al., using the same PGWB questionnaires we have used. Grossi and coworkers reported gender difference PWB scores in 2011 for the Italian population (PWB score females 74.82 and males 81) and in 2013 for citizens of Milan (PWB score females 78.32 and males 83) [47, 48]. We have reported that in a sample population of people nonparticipating in social and cultural activities, women PWB and resilience scores are lower than that of nonparticipating men [50] Moreover, in 2003, Ruini et al. reported a gender difference in favour of men by assessing well-being by means of different questionnaire [53]. Psychological well-being gender gap in favour of men was also reported by Pinquart and Sörensen in elderly [54] and by Hori and Kamo in their comparison of 33 countries [55] The latter authors as well as Ruini et al. suggest that different socialization and expectations by gender and different role of men and women in society explain gender gap in psychological well-being.

Biological factors such as hormones, neurotransmitter, and cytokines have been associated to well-being, differently in men and women [56–61]. Taking into account that, in the present work, we examined a female population with mean age 69,78 ± 6,980 living in the Metropolitan area of city of Naples, located in the South of Italy, we cannot exclude that the PWB gender gap mainly reflects women's “traditional” social role in this area.

According to Havighurst “activity theory,” higher levels of participation in social and leisure activities, and role replacement when roles must be relinquished, promote well-being in older adults [62]. Thus, to achieve a healthy aging it is crucial to have equal opportunities for health, follow healthy diets, maintain social relations, participate in meaningful activities, and enjoy financial security. Participation in social and leisure activities means being willing to reach people, to stay connected, to keep learning, and to be curios, in one word to stay alive [17–23]. Social participation is closely linked to self-esteem, life satisfaction, and mental health status, which makes it a very important factor for quality of life. Engagement with community activities, friendships, and meaningful volunteer work are perceived as strategies for maintaining social participation, especially for people with a chronic disease [9]. Thus, encouraging participation in social and cultural activities could be a key tool to fight social isolation and its health detrimental outcomes.

Our results are in line with data in the literature showing positive association between engaging in leisure and well-being [17–23, 47–63]. In addition, some intervention studies seem to strengthen this observation. In particular, through interventions focused on the development of positive emotions, it is possible to improve well-being and reduce disability in the general population, and in most, if not all, mental disorders. These data indicate that well-being can be modified and that leisure and social activities may be affordable tools to improve well-being [64–72]. Moreover, data coming from research on “happiness genes,” suggesting a genetic root of happiness/well-being, do not rule out gene-environment interaction on the expression “happiness genes.” Availability and access to cultural and social activities are a key element of healthy environment and especially of urban environment [73–75].

Social isolation has been associated with malnutrition in older adults. Since eating is a social event, social isolation can have a negative effect on nutrition, and thus we speculated that social and cultural participation might influence adherence to diet [76–78]. Adequate nutrition is a key factor to healthy aging and to preventing disease onset; nevertheless eating appropriately and, even more, following a diet or a nutritional regimen are never an easy task. Motivations are important factors to eat healthy or to stay on a diet, and they change with age. In >60 subjects, dieting, by-and-large required by health problem, is perceived as a punishment. Social and cultural participation, fighting social isolation, may help >60 to follow healthier life styles, among which is healthy eating, or to accept more “easily” to face stressful situation, like being forced to diet. The results presented here suggest that >60 subjects, in particular females, participating in cultural and social activities, apparently “accept” diet or nutritional regimen better then NP subjects as it is shown by an overall higher score in both PWB and resilience. The higher PWB score observed in P females >60 following a diet deserves, in our view, a special consideration. Pampel in 2012 reported a more consistent association between cultural activities and low body weight in the Western country than elsewhere and that the relationship emerges more consistently for women than men [79].

Subjective well-being significantly correlates with high self-esteem, and self-esteem shares significant variance in both mental well-being and happiness. Self-esteem has been found to be the most dominant and powerful predictor of happiness. Quoting Mann, “Indeed, while low self-esteem leads to maladjustment, positive self-esteem, internal standards and aspirations actively seem to contribute to ‘well-being'” [80]. Body image bears relationship to self-esteem and psychosocial adjustment (e.g., eating disturbances, depression, social anxiety, and sexual functioning) [81]. The association between body image and women's mental and physical health has been investigated with mainly focusing on young women's appearance concerns. However, in the “aging society” body concerns are becoming an issue also for older women, because of age-related changes in both appearance and functioning. In particular, aesthetic appearance is becoming relevant to older women and may lead some women to feel that their bodies are inadequate or lacking. Because of the association between beauty and youth, women lose their social value simply by growing old [82, 83]. The ideal of a thinner body image persists in older adult females, as also suggested by the observation that higher BMI predicts lower psychological well-being only among women. Moreover, body-image concerns are significant to self-esteem in older adulthood [84–86].

Conversely, improvements in body image are related to improvements in self-esteem and psychological well-being [87, 88]. On this basis, we cannot rule out that an “esthetic element” may play a role in the higher score in PWB and resilience reported by this sample of over 60 women participating in cultural and social activities and dieting, independently and far beyond health consciousness.

## 5. Conclusion

The aim of the study presented here was to assess subjective well-being in a sample of residents of the Metropolitan area of Naples, when the city is going through a very difficult time of its long history. To our knowledge, this is the first survey on this topic, and our data represent a suggestive baseline.

The present study has been designed to explore the possible association between cultural and social participation and well-being, which our results apparently support. Much larger and more in-depth studies than ours failed to find a causal link between cultural and social participation and well-being and health [29–89]. However, the association is well-documented and apparently is so appealing that several governments include engagement in cultural and social activities among their strategies to improve well-being and health [90–92]. Since welfare costs are one of the major sources of public finance deficits in the EU, investing in “cultural welfare,” an affordable health preventive and promoting strategy for healthy living and aging, could result in a substantial saving of public resources [93].

## Figures and Tables

**Table 1:** Comparison of the six dimensions of PWB, between females and males. Results are reported as mean ± SD. *P* value has been calculated by Student's *t*-test between each two groups.

PWB dimensions	Females	Males	*P* value
Anxiety	3,34 ± 1,311	3,63 ± 1,19	0,003
Vitality (positive)	3,16 ± 1,229	3,18 ± 1,191	0,835
Depressive mood	3,10 ± 1,237	3,44 ± 1,161	0,0004
Self-control	2,71 ± 1,293	3,14 ± 1,358	<0,0001
Positive WB	2,59 ± 1,139	2,84 ± 1,238	0,008
Vitality (negative)	3,03 ± 1,103	3,21 ± 1,16	0,0423

**Table 2:** *Panel a*: comparison of PWB and resilience scores between normal weight and obese all > 60 subjects. Results are reported as mean ± SD. *P* value has been calculated by Student's *t*-test between each two groups. *Panel b*: comparison of PWB and resilience scores between normal weight and obese > 60 Females results are reported as mean ± SD. *P* value has been calculated by Student's *t*-test between each two groups. *Panel c*: comparison of PWB and resilience scores between normal weight and obese > 60 males. Results are reported as mean ± SD. *P* value has been calculated by Student's *t*-test between each two groups.

	BMI categories	All > 60					
		BMI	*P* value	Resilience	*P* value	PWB	*P* value
a	Normal weight	22,57 ± 1,77	<0,0001	6,09 ± 1,52	NS	70,11 ± 18,48	NS
	Obesity	32,79 ± 3,06		5,85 ± 1,89		66,99 ± 23,44	

	BMI categories	Females > 60					
		BMI	*P* value	Resilience	*P* value	PWB	*P* value

b	Normal weight	22,33 ± 1,7	<0,0001	6,25 ± 1,45	<0,05	69,63 ± 16,95	0,0093
	Obesity	33,72 ± 3,4		5,36 ± 2,19		55,61 ± 22,18	

	BMI categories	Males > 60					
		BMI	*P* value	Resilience	*P* value	PWB	*P* value

c	Normal weight	23,22 ± 1,23	<0,0001	5,98 ± 1,54	NS	72,15 ± 18,89	NS
	Obesity	31,75 ± 1,76		6,17 ± 1,55		72,86 ± 21,78	

**Table 3:** The table depicts the number of >60 male and female subjects distributed according to self-reported diagnosed diseases.

BMI distribution	Females > 60					Males > 60				
	Diabetes	Hypertension	Obesity	CVD	Depression	Diabetes	Hypertension	Obesity	CVD	Depression
Normal weight	9	32	1	23	5	17	28	1	45	7
Overweight	12	31	2	16	9	21	38	2	56	7
Obesity	6	16	16	14	8	10	19	16	20	3

**Table 4:** *Panel a*: comparison of BMI, PWB, and resilience between >60 P and NP. Results are reported as mean ± SD. *P* value has been calculated by Student's *t*-test between each two groups. *Panel b*: comparison of BMI, PWB, and resilience between >60 P and NP Females. Results are reported as mean ± SD. *P* value has been calculated by Student's *t*-test between each two groups. *Panel c*: comparison of BMI, PWB, and resilience between >60 P and NP males. Results are reported as mean ± SD. *P* value has been calculated by Student's *t*-test between each two groups.

	>60 all	BMI	*P* value	Resilience	*P* value	PWB	*P* value
a	P	25,8 ± 3,932	0,004	6,07 ± 1,581	<0,0001	70,53 ± 17,98	<0,0001
	NP	27,1 ± 4,21		5,14 ± 1,83		58,95 ± 23,2	

	>60 males	BMI	*P* value	Resilience	*P* value	PWB	*P* value

b	P	26,18 ± 3,31	NS	6,02 ± 1,53	0,009	73,05 ± 16,93	0,015
	NP	26,6 ± 3,30		5,41 ± 1,64		65,38 ± 18,67	

	>60 females	BMI	*P* value	Resilience	*P* value	PWB	*P* value

c	P	25,25 ± 4,36	0,008	6,11 ± 1,53	<0,0001	68,16 ± 18,67	<0,0001
	NP	27,42 ± 5,25		4,75 ± 2,02		50,31 ± 20,48	

**Table 5:** *Panel a*: BMI, PWB, and resilience in >60 subjects following or nonfollowing a diet in relation to participation to cultural and social activities. Results are reported as mean ± SD. *P* value has been calculated by Student's *t*-test between each two groups. *Panel b*: BMI, PWB, and resilience in females >60 following or nonfollowing a diet in relation to participation in cultural and social activities. Results are reported as mean ± SD. *P* value has been calculated by Student's *t*-test between each two groups. *Panel c*: BMI, PWB, and resilience in males >60 following or nonfollowing a diet in relation to participation in cultural and social activities. Results are reported as mean ± SD. *P* value has been calculated by Student's *t*-test between each two groups.

		All > 60							
		Yes diet				No diet			
		PWB	*P* value	Resilience	*P* value	PWB	*P* value	Resilience	*P* value
a	P	70,79 ± 18,56	0,001	6,10 ± 1,58	0,02	70,08 ± 17,92	0,002	6,04 ± 1,58	<0,001
	NP	57,73 ± 22,47		5,43 ± 1,66		59,78 ± 23,69		5,05 ± 1,92	

		Males > 60							
		Yes diet				No diet			
		PWB	*P* value	Resilience	*P* value	PWB	*P* value	Resilience	*P* value

b	P	73,33 ± 18,59	0,028	6,15 ± 1,57	0,192	72,68 ± 16,34	NS	5,94 ± 1,54	NS
	NP	57,59 ± 26,01		5,59 ± 1,66		71,94 ± 19,83		5,65 ± 1,53	

		Females > 60							
		Yes diet				No diet			
		PWB	*P* value	Resilience	*P* value	PWB	*P* value	Resilience	*P* value

c	P	68,35 ± 18,22	0,013	6,05 ± 1,60	0,043	67,60 ± 19,05	<0,001	6,13 ± 1,63	<0,001
	NP	57,09 ± 20,14		5,24 ± 1,70		43,73 ± 19,27		4,22 ± 2,17	
